# Investigation of risk factors for tunneled hemodialysis catheters dysfunction: competing risk analysis of a tertiary center data

**DOI:** 10.1186/s12882-022-02927-z

**Published:** 2022-09-02

**Authors:** Arash Mohazzab, Morteza Khavanin Zadeh, Paria Dehesh, Neda Abdolvand, Zhaleh Rahimi, Sahar Rahmani

**Affiliations:** 1 Epidemiology Department, School of Public Health, Iran University of Medical Sciences, Tehran, Iran; 2 Reproductive Biotechnology Research Center, Avicenna Research Institute, ACECR, Tehran, Iran; 3 Hasheminejad Kidney Center (HKC), Iran University of Medical Sciences, Tehran, Iran; 4 Department of Information Technology Management, Faculty of Social Sciences and Economics, Alzahra University, Tehran, Iran

**Keywords:** Hemodialysis tunneled catheter, Catheter dysfunction, Competing risk analysis, Catheter thrombosis, Catheter infection

## Abstract

**Background:**

Hemodialysis tunneled catheters are prone to failure due to infection or thrombosis. Prediction of catheter dysfunction chance and finding the predisposing risk factors might help clinicians to prolong proper catheter function. The multidimensional mechanism of failures following infection or thrombosis needs a multivariable and comprehensive analytic approach.

**Methods:**

A longitudinal cross-sectional study was implemented on 1048 patients admitted for the first hemodialysis tunneled catheterization attempt between 2013 and 2019 in Shahid Hasheminejdad hospital, Tehran, Iran. Patients’ information was extracted from digital and also paper records. Based on their criteria, single and multiple variable analyses were done separately in patients with catheter dysfunction due to thrombosis and infection. T-test and Chi-square test were performed in quantitative and categorical variables, respectively. Competing risk regression was performed under the assumption of proportionality for infection and thrombosis, and the sub-distributional hazard ratios (SHR) were calculated. All statistical inferences were made with a significance level of 0.05.

**Results:**

Four hundred sixty-six patients were enrolled in the analysis based on study criteria. Samples’ mean (SD) age was 54(15.54), and 322 (69.1%) patients were female. Three hundred sixty-five catheter dysfunction cases were observed due to thrombosis 123(26.4%) and infection 242(52%). The Median (range) time to catheter dysfunction event was 243(36–1131) days.

Single variable analysis showed a statistically significant higher proportion of thrombosis in females (OR = 2.66, 95% CI: 1.77–4.00) and younger patients, respectively. Multivariate competing risk regression showed a statistically significant higher risk of thrombosis in females (Sub-distributional hazard (SHR) = 1.81), hypertensive (SHR = 1.82), and more obese patients (BMI SHR = 1.037). A higher risk of infection was calculated in younger (Age SHR = 0.98) and diabetic (SHR = 1.63) patients using the same method.

**Conclusion:**

Female and hypertensive patients are considerably at higher risk of catheter thrombosis, whereas diabetes is the most critical risk factor for infectious catheter dysfunction. Competing risk regression analysis showed a comprehensive result in the assessment of risk factors of catheter dysfunction.

## Introduction

Suitable vascular access is the main concern in chronic hemodialysis patients. Tunneled central venous catheters (CVC) is a flexible tube with prolonged vascular access providing for the management of intravenous medication treatments, fluids, or total parenteral nutrition, repeated blood sampling, and hemodialysis (HD) [1]. CVCs are used as temporary access to the vascular until permanent access to the vascular (arteriovenous fistula [AV F] or arteriovenous graft [AVG]) can be placed [1]. Approximately 80% of newly hemodialysis patients require a CVC because they do not have suitable AFV to use or they have not had permanent access placed before dialysis initiation.

Identification and prevention of CVCs complications in hemodialysis patients (Common complications such as catheter infection and thrombosis) are serious to improving patient care. CVC’s main complications include infection, catheter dysfunction, and central vein obstruction (CVO). Infection associated with hemodialysis catheters is the most serious complication that may cause significant morbidity and mortality, systemic complications, hospitalizations, and considerable costs to the healthcare system [2]. Catheter dysfunction is defined as the lack of ability of a central venous catheter to: (1) doing a single dialysis period without causing recurrent pressure alarms or (2) generate hand over a mean dialysis blood flow of > 300 ml/min on two sequential dialysis periods or deliver a Kt/V of 1.2 in 4 hours or less [3]. Catheter dysfunction may be caused due thrombosis, fibrin sheath obstruction and mechanical complications. Central vein obstruction (CVO) is identified in patients with > 70% stenosis of a central vein by contrast venography, or upper limb edema on the same side, and having a history of a central venous catheter [3]. The incidence rate of CVCs complications is very wide because it can be determined by the definition of complications, patient population, units of measurement, duration of catheterization and follow-up, catheter location, and diagnostic methods [4]. The US Renal Data System delivers guidelines for coding complications of the catheter in hemodialysis patients [5].

Previous research about the variables influencing the complications of the tunneled central venous catheters occurred in hemodialysis patients is shown that the Localization of the catheter into the right internal jugular vein [1], duration of catheterization and diabetes [6] increase the risk of infection and also younger hemodialysis patients have a higher risk of infection [7]. However, the use of anticoagulant agents decreases the risk of thrombosis but is not significant [1]. Since there is a little knowledge about the variables that effect on the time until common complications of CVCs occurs, this study aims to find the effect of several risk factors on the time until thrombosis and infections of the catheter in hemodialysis patients. A better understanding of these factors may lead to having better survival and lower complication in the CVCs.

## Method

### Design and patients

This longitudinal cross-sectional study was implemented to assess the risk factors affecting the function of tunneled central venous catheters that had been fixed for end-stage renal disease (ESRD) patients to perform hemodialysis. The study was conducted in Haeshmei-Nedjad Kidney Center, Tehran, Iran, between June 2019- Feb 2020.

All patients who underwent central venous catheterization for the first time between the years 2015–2019 in the hospital were included in the study if their medical records were completed from the first catheterization episode until its dysfunction, end of follow-up, or end of the study period. Inclusion criteria were the first attempt for tunneled hemodialysis catheterization. All catheterizations were performed ultrasound-guided and rechecked by fluoroscopy to confirm the proper replacement of the catheter in the Cava-atrial junction. Based During the observed period, all patients underwent hemodialysis 2–4 times per week based on the ward protocol, and their catheters were heparin locked by 2500 units after each dialysis session.

Patients who encountered catheter dysfunction in less than 1 months, those who had been lost to follow for catheter dysfunction, and those who were referred for the second or more installation of the venous catheter were excluded from the study. Moreover, any patient with a history of a thrombotic event, thrombotic dysfunction, immunodeficiency, and also recent use of antithrombotic, antibiotics or chemotherapy medication were excluded too. Patients’ information, including demographic, past medical, and current medical situation, was extracted from the hospital information system (HIS) and digital records. In case of missing data (especially in older files), we referred to a paper medical file to complete the data. All patients signed informed consent to allow use their medical information anonymously for research goals.

### Factors and outcomes

Registered demographic information was age, BMI, educational level and residential area. Past medical history of patients such as diabetes mellitus (DM), hypertension, Ischemic Heart disease (IHD) and its related interventions, and cancer, in addition to their clinical and para-clinical information before and after the admission time includes were extracted. Anemia was defined as hemoglobin (Hb) levels < 12.0 g/dL in women and < 13.0 g/dL in men based on WHO definition [8]. Notably, any renal intervention such as the history of hemodialysis and its duration, renal transplantation history, brachial fistula fixation, and location of central venous catheterization were recorded. Patients’ nursing care levels were categorized by Welsh Levels of Care.

The study’s endpoint was defined as catheter replacement due to thrombosis or infection and the time between first and second catheterization.

Thrombosis of the catheter was defined as a significant impairment of the blood flow in the catheter (Blood flow rate (QB) = 150–250 ml/min adjusted based on patients’ weight) upon initiating the dialysis session. Since thrombolytic agents (Such as Urokinase) as not covered by medical insurance, we did not use them in case of catheter thrombosis.

The infected catheter was diagnosed based on the patient’s clinical symptoms (fever or chilling) and subsequent positive blood culture test results from the catheter line or peripheral.

### Statistical analysis

After the descriptive report of variables in the study samples, single and multivariable analyses were performed using STATA software version 16. Single variable analysis was done using the independent sample t-Test and also the Chi-square test for quantitative or categorical variables.

Since the study’s main outcome was the time to reach catheter dysfunction, the multivariate statistical analysis plan was based on survival analysis, and patients who had never experienced dysfunction were considered censored (even if death happened due to non-catheter related reasons). Two separate cox regression models were adjusted to assess the association between factors and two causes of catheter dysfunction (Thrombosis and infection). Proportionality assumptions were assessed for any assumed risk factors before involving in the regression models. Moreover, due to trigonal characteristics of patients’ state (continuously functional catheter, dysfunction by thrombosis, and dysfunction by infection), it seems infection and thrombosis compete for catheterization failure. Then the risk factors effect was evaluated using competing risk regression analysis between these two different outcomes and the sub-distributional hazard ratio was calculated for both infection and thrombosis competing with each other by STATA software. The significance level was considered as 0.05 in univariate analysis. Multivariate analyses were performed in the backward method. Variables were kept in the model if the univariate *P*-value was < 0.2. However, some variables were included in the model regardless of the *p*-value in the case of clinical intuition.

## Results

**Fig. 1:**
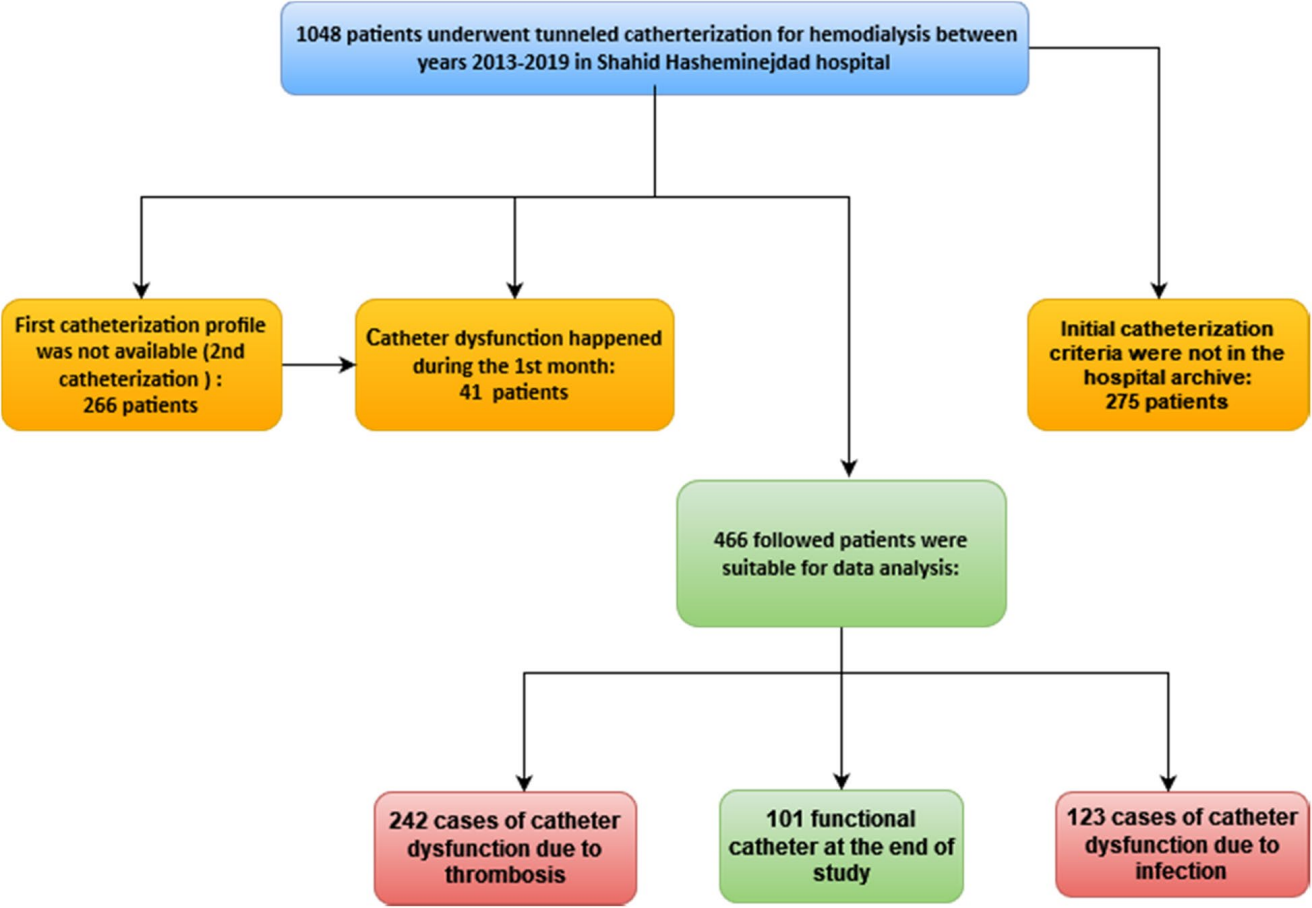
Patients’ flow diagram

Throughout 1048 central venous catheterization cases in the study period, 466 patients were completely eligible for analysis (Fig. 1). Within these cases, 242(52%) patients experienced thrombosis, 123(23.4%) dysfunction happened due to infection, and the remaining catheter continued working into the end of follow-up period. The median (range) time for proper catheter working is 243(36–1131) days in total. This median (range) time were 217(36–1075), 247(37–1060), 486(36–1131) in thrombosed, infected and working catheter respectively.

There was no case of death before catheter dysfunction. Jugular catheterization was performed in 95.5% of patients whereas sub-clavian and femoral catheter was fixed in 2.4 and 2.1% cases respectively. Patients’ care level was 6.9, 50.5, 34.7 and 7.9% from level 1 to 4 respectively. Patients’ clinical characteristics are described in Table 1.

**Table 1:** Patents’ clinical characteristics

	Male (144)	Female (322)	Total
**Age**	52.95 (17.99)	55.74 (14.25)	54 (15.54)
**Menopause**	–	227 (70.5%)	–
**DM**	51 (35.4%)	69 (21.4%)	120 (25.8%)
**HTN**	86 (59.7%)	95 (29.5%)	181 (38.8%)
**CVA**	10 (6.9%)	9 (2.8%)	19 (4.1%)
**Anemia**	65 (91.5%)	71 (77.2%)	136 (83.4%)
**Functional Catheter time**	236 (37–1131)	261 (36–1064)	243 (36–1131)

Data are demonstrated as Mean (SD), and number (percentage)

Single variable analysis showed that older patients’ are at higher risk of thrombosis and lower risk of infection (Table 2), although these differences were not observed when age is adjusted with other variables (Table 3). The female gender showed a considerably higher risk of thrombosis both in single and adjusted analysis (Odds ratio (OR) = 2.66, 95% Confidence interval (CI):1.77–4.00), whereas there was no relationship between gender and infection (Tables 2 and 3).

**Table 2:** Univariate analysis of patients’ risk factors for catheter dysfunction, comparing patients with infection or thrombosis event versus no event

	Infection			Thrombosis		
	Yes 123 (26.3%)	No 343 (73.6)	***P***-value	Yes 242 (52%)	No 224 (48%)	***P***-value
**Age**^**a**^	51.24 (17.01)	56.18 (14.75)	0.005	56.68 (14.02)	52.93 (16.84)	0.012
**Gender (Female)**^**b**^	79 (64.2%)	243 (70.8%)	0.173	191 (78.9%)	131 (59.3%)	0 < 0.001
**BMI**^**a**^ **(Kg/m**^**2**^**)**	26.4 (4.87)	27.08 (6.1)	0.393	27.63 (5.88)	26.37 (5.58)	0.127
**INR**^**a**^	–	–	–	1.12 (0.24)	1.08 (0.12)	0.186
**Anemia**^**b**^	32 (30.6%)	15 (14.9%)	0.388	14 (16.9%)	13 (16.9%)	0.998
**Diabetes**^**b**^	34 (27.6%)	86 (25.1%)	0.576	54 (22.3%)	64 (29%)	0.101
**Hypertention**^**b**^	48 (39%)	133 (38.8%)	0.961	95 (39.3%)	86 (38.4%)	0.783

^a^Data are demonstrated as Mean (SD), and analyzed using independent T test

^b^Data are demonstrated as number (percentage), and analyzed using Chi square test

**Table 3:** Competing risk analysis for thrombosis

	Univariate			Multivariate		
Factor	SHR^**a**^	95% Confidence interval	***P***-value	SHR	95% Confidence interval	***P***-value
**Age**	1.009	0.99–1.02	0.063	1.01	1–1.02	0.047
**Gender (Female = 1)**	1.71	1.23–2.39	0.001	1.82	1.291–2.571	0.001
**Education**	0.84	0.60–1.17	0.310			
**BMI (Kg/m**^**2**^**)**	1.033	0.99–1.069	0.053	1.037	1.001–1.074	0.040
**Systolic Blood pressure**	1.002	0.99–1.009	0.56			
**Diastolic Blood pressure**	1.031	0.999–1.027	0.076	1.009	0.994–1.025	0.212
**Diabetes**	0.95	0.689–1.328	0.794	0.66	0.451–0.972	0.035
**Hypertention**	1.40	1.04–1.90	0.026	1.81	1.290–2.559	0.001
**CABG**^**b**^	1.55	0.882–2.781	0.125	1.69	0.889–3.215	0.109
**CVA**^**c**^	0.89	0.45–1.73	0.732			
**IHD**^**d**^	1.0840	0.277–4.24	0.907			
**Severe anemia**^**e**^	0.909	0.573–1.442	0.686			
**Hemoglobin (g/dL)**	1.02	0.938–1.1240.566	0.922			
**Creatinin**	0.945	0.883–1.012	0.111	0.974	0.902–1.053	0.516

Variables were included in the multivariate analysis if the univariate *P*-vale< 0.2; Therefore, Education, Systolic blood pressure, CABG, CVA, IHD, Severe anemia, and Hemoglobin level were excluded from the multivariate analysis

^a^Sub-distributional hazard ratio

^b^Coronary artery bypass graft

^c^Cardiovascular Accident

^d^Ischemic heart disease

^e^Severe anemia was defined as Hb < 8 based on WHO criteria

Multivariate regression for thrombosis, which competed by infection, demonstrated that female gender and hypertension predispose patients to thrombosis while DM is associated with a reduced risk of it. On the other hand, higher age and lower diastolic pressure are associated with a lower chance of infection in competing risk of thrombosis since diabetes makes patients more susceptible to infection (Table 4).

**Table 4:** Competing risk analysis for infection

	Univariate			Multivariate		
Factor	SHR^**a**^	95% Confidence interval	***P***-value	SHR	95% Confidence interval	***P***-value
**Age (years)**	0.98	0.971–0.995	0.006	0.982	0.968–0.994	0.004
**Gender (Female = 1)**	0.727	0.485–1.088	0.121	0.726	0.474–1.18	0.140
**Education**	1.275	0.773–2.103	0.341			
**BMI (Kg/m**^**2**^**)**	0.985	0.943–1.028				
**Systolic Blood pressure (mm Hg)**	0.99	0.98–1.002	0.217			
**Diastolic Blood pressure (mm Hg)**	0.985	0.970–0.999	0.045	0.985	0.971–0.999	0.041
**Diabetes**	1.172	0.771–1.78	0.457	1.633	1.03–2.58	0.036
**Hypertention**	0.856	0.572–1.27	0.448	0.704	0.459–1.07	0.107
**CABG**^**b**^	0.681	0.241–1.920	0.468			
**CVA**^**c**^	1.100	0.503–2.405	0.811			
**IHD**^**d**^	0.999	0.313–3.193	0.970			
**Severe anemia**^**e**^	1.22	0.675–2.22	0.502			
**Hemoglobin (g/dl)**	1.002	0.877–1.145	0.970			
**Creatinin**	1.109	0.971–1.069	0.435			

Variables were included in the multivariate analysis if the univariate *P*-vale< 0.2; Therefore, BMI, Education, Systolic blood pressure, CABG, CVA, IHD, Severe anemia, Hemoglobin and Creatinine level were excluded from the multivariate analysis

^a^Sub-distributional hazard ratio

^b^Coronary artery bypass graft

^c^Cardiovascular accident

^d^Ischemic heart disease

^e^Severe anemia was defined as Hb < 8 based on WHO criteria

## Discussion

This single-center study tried to investigate many predisposing risk factors for catheter dysfunction with about 116,000 days follow-up through two types of a regression model. Findings proposed that female and hypertensive patients besides more obese individuals, are considerably higher at risk of catheter thrombosis. In contrast, diabetes is the most critical risk factor for infectious catheter dysfunction. The result of the competing risk model is more reliable due to the best adjustment.

Previous studies reported different rates of thrombosis. A similar percentage were observed in 46.7% of cases with hemodialysis catheter in the Sahli study [6]. In a study in Croatia, this rate was 16.8 in 1 year following up of patients with the central catheter for dialysis [9]. Develter et al. reported a 12% rate for thrombosis as a catheter dysfunction cause which is considerably lower than our results. No comorbidity was found as a predictor [1]. The difference in observed thrombosis rate might be affected by the length of patient follow-up and also the use of antithrombotic/thrombolytic protocols since Hemmelgarn et al. and also ward et al. reported a significant difference between recombinant tissue-plasminogen activator and heparin lock solution in thrombosis rate [10, 11].

The infection rate reported widely from 6 to 29% in the various surgical settings and also patients population [6, 12–14] which is compatible with our findings of 26.4%. Sometimes the infection is the main reason for catheter removal, which differs from our results. This variety might be due to patients’ care level distribution and exposure to infectious organisms. In our study, care level 1 is directly associated with a lower risk of infection.

Multivariate analysis to assess the risk factors of thrombosis and also infection in patients with tunneled central catheters is performed frequently using Cox or logistic regression, but most studies used these methods for each endpoint separately [4, 6, 11, 15]. A cohort study compared the risk factors of death rate in tunneled hemodialysis catheter and aterio-venous fistula, which is different in final outcomes [2]. These risk calculation methods assume that thrombosis and infection are independent, and there is no mutual risk factor that confounds or interacts between these two endpoints outcomes. This assumption may be a risk of bias because it seems thrombosis or infection compete with each other in catheter dysfunction, and this competition should be considered in any factor analysis [16]. Therefore competing risk analysis is the better selection for adjusting the risk of different variables in the multivariate analysis approach.

On the base of the author’s knowledge, the competing risk regression model has not been used for risk factor analysis in hemodialysis tunneled catheters but it was used recently for peritoneal dialysis-associated factors [17].

Patients’ age in our study is lower compared with most previous studies especially compared in developed countries [1, 11, 12, 18] but this might be due to earlier incidence of end-stage renal disease in developing country which observed similarly in some reported articles [6, 13, 15]. Competing risk analyses have shown a higher risk of infection in younger patients, which is compatible with the results of Wang et al. study [15] but against Sahli et al. results which rejected the role of age in this outcome. The patients’ care level and outdoor activity should be considered to infer this association. On the other hand, age is associated with a higher chance of thrombosis adjusted with other risk factors, which was reported previously by Ward et al. [11].

A higher rate of thrombosis in females was reported considerably by Ward in the direction of our results. However, Pasara et al. reported a higher mortality rate in men due to catheter dysfunction [9]. Our results or previous studies did not find any association between infection and gender.

Our results show hypertensive patients have a considerably higher risk of thrombosis SHR = 1.4. Equivalent results were reported in the Ward study, where higher mean blood pressure at the time of dialysis is reported as a risk factor for thrombosis. Unfortunately, this variable is not available in our database, although to quantify the effect of hypertension on thrombosis, we include diastolic blood pressure at the time of catheter insertion in a multivariate competing regression model, but the results were not statistically significant. Pasara reported a lower chance of death in hemodialysis patients with previous hypertension history.

Higher BMI and obesity are frequently mentioned as risk factors for catheter thrombosis, and this is entirely compatible with the current study’s result [19].

The current study introduces new methods of risk factor analysis for hemodialysis catheter dysfunction whit competing for risk calculation which is recommended to be considered for future studies. Single-center study population made the intervention protocol more homogenous, although this might reduce its external validity. The study’s main limitation is its retrospective nature, leading to missing data, loss of patient follow-up, and a lack of good quality laboratory data registry.

## Data Availability

The datasets analyzed during the current study are not publicly available because it is a part of an extensive university-affiliated database and the raw data’s intellectual property belongs to the Iran University of medical science. However, it is available from the corresponding author on reasonable request.

## References

[1] Develter W, De Cubber A, Van Biesen W, Vanholder R, Lameire N (2005). Survival and complications of indwelling venous catheters for permanent use in hemodialysis patients. Artif Organs.

[2] O’grady NP, Alexander M, Burns LA, Dellinger EP, Garland J, Heard SO (2011). Guidelines for the prevention of intravascular catheter-related infections. Clin Infect Dis.

[3] Allon M, Brouwer-Maier DJ, Abreo K, Baskin KM, Bregel K, Chand DH (2018). Recommended clinical trial end points for dialysis catheters. Clin J Am Soc Nephrol.

[4] Boersma R, Jie K-S, Verbon A, Van Pampus E, Schouten H (2008). Thrombotic and infectious complications of central venous catheters in patients with hematological malignancies. Ann Oncol.

[5] Renal DU (2013). System, USRDS 2013 annual data report: atlas of chronic kidney disease and end-stage Renal disease in the United States, National Institutes of Health, National Institute of Diabetes and Digestive and Kidney Diseases.

[6] Sahli F, Feidjel R, Laalaoui R (2017). Hemodialysis catheter-related infection: rates, risk factors and pathogens. J Infect Public Health.

[7] Napalkov P, Felici DM, Chu LK, Jacobs JR, Begelman SM (2013). Incidence of catheter-related complications in patients with central venous or hemodialysis catheters: a health care claims database analysis. BMC Cardiovasc Disord.

[8] WHO (2011). Haemoglobin concentrations for the diagnosis of anaemia and assessment of severity. Vitamin and mineral nutrition information system Geneva: World Health Organization, 2011.

[9] Pašara V, Maksimović B, Gunjača M, Mihovilović K, Lončar A, Kudumija B (2016). Tunnelled haemodialysis catheter and haemodialysis outcomes: a retrospective cohort study in Zagreb, Croatia. BMJ Open.

[10] Hemmelgarn BR, Moist LM, Lok CE, Tonelli M, Manns BJ, Holden RM (2011). Prevention of Dialysis catheter malfunction with recombinant tissue plasminogen activator. N Engl J Med.

[11] Ward DR, Moist LM, MacRae JM, Scott-Douglas N, Zhang J, Tonelli M (2014). Risk factors associated with hemodialysis central venous catheter malfunction; a retrospective analysis of a randomized controlled trial. Can J Kidney Health Dis.

[12] Jean G, Charra B, Chazot C, Vanel T, Terrat JC, Hurot JM (2001). Long-term outcome of permanent hemodialysis catheters: a controlled study. Blood Purif.

[13] Fox J, Joubert G, Loggenberg E (2019). Tunnelled haemodialysis catheters in Central Free State: epidemiology and complications. SA J Radiol.

[14] Ekbal NJ, Swift PA, Chalisey A, Steele M, Makanjuola D, Chemla E (2008). Hemodialysis access-related survival and morbidity in an elderly population in south West Thames, UK. Hemodial Int.

[15] Wang K, Wang P, Liang X, Lu X, Liu Z (2015). Epidemiology of haemodialysis catheter complications: a survey of 865 dialysis patients from 14 haemodialysis centres in Henan province in China. BMJ Open.

[16] Haller B, Schmidt G, Ulm K (2013). Applying competing risks regression models: an overview. Lifetime Data Anal.

[17] Chen H-L, Tarng D-C, Huang L-H (2019). Risk factors associated with outcomes of peritoneal dialysis in Taiwan: an analysis using a competing risk model. Medicine (Baltimore).

[18] Worth LJ, Seymour JF, Slavin MA (2009). Infective and thrombotic complications of central venous catheters in patients with hematological malignancy: prospective evaluation of nontunneled devices. Supportive Care Cancer.

[19] Leung A, Heal C, Perera M, Pretorius C (2015). A systematic review of patient-related risk factors for catheter-related thrombosis. J Thromb Thrombolysis.

